# Factors associated with response and remission from depression at 6-months of treatment in a retrospective cohort treated within an integrated care program

**DOI:** 10.1186/s12913-021-06729-1

**Published:** 2021-07-16

**Authors:** Jessica Jeffrey, Alex Klomhaus, Hilary Aralis, Wendy Barrera, Shanna Rosenberg, Mark Grossman, Patricia Lester

**Affiliations:** 1 Division of Population Behavioral Health, Department of Psychiatry and Biobehavioral Sciences, Jane and Terry Semel Institute for Neuroscience and Human Behavior, UCLA, 760 Westwood Plaza, A7-372A, Los Angeles, CA 90024 USA; 2 Departments of Medicine and Pediatrics, UCLA Health, Los Angeles, CA USA

**Keywords:** Integrated care, Traumatic stress, Co-morbidities, Depression treatment, Screening, Suicidal ideation

## Abstract

**Background:**

Depression causes significant morbidity, which impacts mental health, overall general health outcomes, everyday functioning and quality of life. This study aims to contribute to knowledge in the field through enhanced understanding of factors that influence depression response and remission, with consideration for design of treatment services to optimize depression outcomes within integrated care programs.

**Methods:**

Using routine behavioral health screening and electronic health record data, we identified a retrospective cohort consisting of 615 adult patients receiving depression treatment within an integrated care program. Cohort member Patient Health Questionnaire (PHQ-9) data was analyzed for the 6 months following initiation of treatment. Multinomial regression models were estimated to identify factors associated with depression treatment response (PHQ-9 < 10) and remission (PHQ-9 < 5).

**Results:**

At 6 months, 47% of patients demonstrated treatment response and 16% demonstrated remission. Baseline trauma symptoms and suicidal ideation were significantly associated with decreased odds of achieving remission (Odds Ratio (95% CI) [OR] = 0.45 (0.23, 0.88) and OR = 0.49 (0.29, 0.82), respectively). In fully adjusted models, baseline suicidal ideation remained significant (OR = 0.53 (0.31, 0.89)) and some evidence of an association persisted for baseline trauma symptoms (OR = 0.51 (0.25, 1.01)).

**Conclusions:**

After controlling for baseline depression symptoms, the presence of suicidal ideation is associated with reduced likelihood of remission. Increased understanding of factors associated with depression treatment outcomes may be employed to help guide the delivery and design of clinical services. Alongside routine screening for co-morbid anxiety, suicidal ideation and traumatic stress should be assessed and considered when designing depression treatment services.

## Background

Depression is common, with 17.3 million adults within the United States (U.S.), and over 264 million people worldwide experiencing depression [1, 2]. Depression is the leading cause of disability worldwide [2]. Depression is often a chronic disease, with frequent relapses and recurrences over the lifetime [3]. Depression causes significant morbidity, which impacts mental health, overall general health outcomes, everyday functioning [4–6] and quality of life [6]. Depression is associated with medically unexplained physical symptoms such as pain and fatigue [7, 8], and poorer prognosis of chronic medical conditions such as diabetes, asthma, and hypertension [4, 9]. In the U.S., depression is the leading cause of suicide [10] and it has a significant impact on the economy in terms of decreased work productivity, direct medical costs [11], and suicide-related mortality costs [5].

Given the burden of depression and the treatability of this condition, routine screening, when adequate systems are in place to ensure accurate diagnosis and follow-up [12], and monitoring of depression treatment outcomes is recommended [13]. Within the U.S., both private and public payers require and incentivize depression screening and documentation of depression remission. Thus, it is important for providers and health systems to understand patient-related and systems-level factors associated with depression remission and response rates in treatment settings, as increased understanding of these factors will help to guide the delivery and design of services to help patients achieve remission from depression symptoms.

Population-based behavioral health programs are being implemented within primary care settings to facilitate the identification and treatment of depression [14]. Behavioral health integration within primary care is associated with significant improvement in depression outcomes compared with usual care [15–17]. Behavioral Health Associates (BHA) is an integrated care program operated as part of UCLA Health. BHA incorporates several core principles of the IMPACT model [18], including evidence-based care, patient-centered care, and measurement-based-treatment-to-target based on standardized symptom assessments [14]. Screening and monitoring of treatment occur through use of the psychometrically-validated, self-report Patient Health Questionnaire-9 (PHQ-9) rating scale, given its high sensitivity and specificity as well as responsiveness to change in symptom severity over time [19–21].

Previous research has examined factors that influence depression response and remission treatment outcomes. Studies examining stepped treatment with antidepressant medications have revealed depression remission is informed by such factors as patient demographics and health co-morbidities [22]. For instance, Gaynes et al. [22] reported improved depression outcomes for patients who are female, employed, and have higher education and income. On the other hand, behavioral health comorbidities such as anxiety and substance use, medical comorbidities, and lower levels of functioning at baseline were associated with lower rates of depression remission [22]. Substances may be used as a coping mechanism by patients with behavioral health symptoms [23, 24]. Importantly, residual subsyndromal symptoms of depression increase risk of relapse and recurrence [3].

Primary care patients’ depression treatment outcomes may also be impacted by previous traumatic experiences. Bomyea et al. [25] report that 65–88% of civilians in primary care have been exposed to a traumatic event. Within the UK, the Adult Psychiatric Morbidity Survey, revealed rates of positive screening for posttraumatic stress disorder were 12.6% in females compared and 3.6% in males [26]. With the high prevalence of trauma exposure reported in primary care populations, and the additional association of trauma symptoms with depression and chronic medical conditions [27], it is important to understand how these symptoms impact depression treatment outcomes. Given the increased implementation of screenings for adverse childhood experiences within primary care settings, it may be expected that more traumatic experiences as well as traumatic stress symptoms will be increasingly identified in primary care patients and that this exposure will need to be taken into account in behavioral health treatment planning [28, 29]. Childhood trauma has been associated with poorer depression outcomes and greater risk of relapse [30–32].

This study aims to contribute to knowledge in the field through enhanced understanding of factors that influence depression response and remission with the goal of informing the design of treatment services to optimally improve depression outcomes within integrated care programs. Given the increasing emphasis on quality metrics as they pertain to depression response and remission, this paper examines factors associated with treatment response (defined as PHQ-9 < 10) and remission (defined as PHQ-9 < 5) at 6-months of treatment within a behavioral health program integrated within primary care, BHA [14]. In addition to the treatment outcomes of response and remission, we also examine factors associated with reliable improvement based on calculation and application of a reliable change index. We hypothesize that improved depression treatment outcomes will be associated with fewer baseline behavioral health comorbid symptoms of anxiety, substance use, and post-traumatic stress. We also hypothesize that lack of endorsement of suicidal ideation will be associated with improved treatment outcomes.

## Methods

### Context

The BHA program was developed by adapting features of the AIMS Center Collaborative Care model to support delivery of behavioral health services to primary care patients within a large, urban medical center. The program includes team-based care with psychiatrists and master’s-level therapists in clinics co-located within primary care settings, provision of short-term evidence-based therapies, care coordination, and measurement-based care protocols [14]. E-consultation, with the option for telephone consultation, is provided to primary care providers by psychiatrists. BHA has been in operation since November 2012 and has since expanded to 13 urban primary care locations within the Los Angeles area.

### Data sources

Behavioral health assessment and tracking of results is a core component of collaborative care which enables providers to assess treatment progress and effectively guide care. To accomplish this goal, BHA leadership implemented the UCLA Behavioral Health Checkup (BHC) assessment tool beginning in February 2014 [33]. The BHC is a cloud-based behavioral health assessment and clinician decision-making tool that provides real-time results along with clinical interpretation to inform delivery of care. The BHC consists of psychometrically-validated, patient self-report measures available in the public domain. BHC assessments are completed on a tablet in the clinic waiting room prior to a behavioral health intake appointment and again at 3-month increments and provide an integrated registry within the electronic health record (EHR). To augment BHC data, patient characteristics and visit data were obtained from UCLA’s EHR. All data pertained to patients seen at a BHA clinic for treatment of behavioral health symptoms.

### Study population

**Fig. 1:**
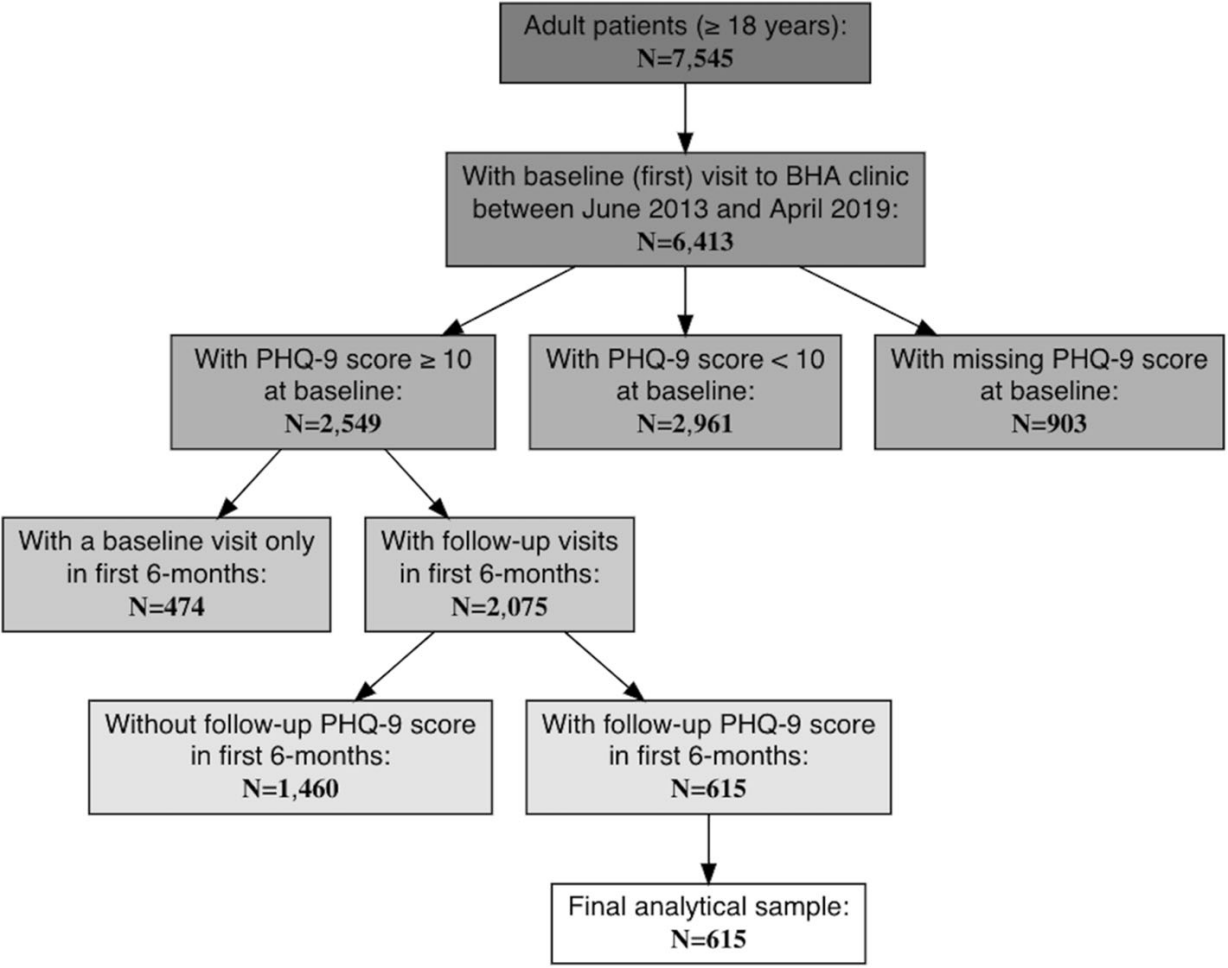
Flowchart describing derivation of the final analytical sample

The study sample comprised patients age 18 and older who were screened for depression symptoms on their baseline (first) visit at a BHA clinic between June 2013 and April 2019 and at least once more within 6 months of their baseline visit. Patients were included in the analytical sample if they reported elevated depression symptoms (PHQ-9 ≥ 10) at their baseline visit, initiated treatment on or before April 27, 2019, and had multiple PHQ-9 scores recorded in the first 6 months of treatment (relative to baseline). Our final analytical sample consists of *N* = 615 patients. Figure 1 can be referenced for further details regarding the application of exclusion criteria.

### Measures

#### Mental health and substance use

Self-reported mental health and substance use measures were collected from patients during clinic visits through the BHC. Table 1 presents the full list of measures and cut-offs used in analyses. We chose to define suicidal ideation by dichotomizing item 9 of the PHQ-9, rather than treating item 9 as an ordinal variable. This approach has practical utility to clinicians who have received guidance to deliver measurement-based care wherein a score of > 0 on this item triggers comprehensive evaluation and follow-up. Additionally, previous research has established that any score > 0 on the PHQ-9 item 9 is significantly associated with the hazard of suicide attempts [41]. In our sample, 40% of patients reported a score > 0 on item 9 and less than 15% reported a score of 2 or 3. A reliable change index was calculated using the approach described by [42]. Specifically, we used the internal reliability estimate (Cronbach’s *α*) of 0.89 for the PHQ-9 reported in the original validation study [20] and the pre-treatment (baseline) standard deviation from the current study (6.414; calculated using the sample prior to excluding patients with PHQ-9 Total Score < 10). This resulted in PHQ-9 score reductions from pre- to post-treatment of ≥6 being indicative of reliable change.

**Table 1:** Self-reported measures assessing mental health and substance use

Measure	Domain	Number of Items	Period Assessed	Response Scale	Scoring Algorithm	Cutoff Score	Internal Consistency
**Mental Health**							
Patient Health Questionnaire-9 (PHQ-9) [20]	Depression	9	Past two weeks	Likert, 0 (“Not at all”) to 3 (“Nearly every day”)	PHQ-9 Total Score = sum of all nine items (score range, 0 to 27) PHQ-8 Total Score = sum of items 1 to 8 (score range, 0 to 24)^a^	PHQ-9 Total Score ≥ 10, moderate-to-severe depression symptoms [34]	α = 0.68 (α = 0.86 prior to excluding patients with PHQ-9 Total Score < 10)
Patient Health Questionnaire-9 (PHQ-9) – Item 9 [20]	Suicidal Ideation	1	Past two weeks	Likert, 0 (“Not at all”) to 3 (“Nearly every day”)	PHQ-9 Item 9 = sum of one item (score range, 0–3)	PHQ-9 Item 9 ≥ 1, any frequency of suicidal ideation	N/A
Generalized Anxiety Disorder-7 (GAD-7) [35]	Anxiety	7	Past two weeks	Likert, 0 (“Not at all”) to 3 (“Nearly every day”)	Total Score = sum of all seven items (score range, 0 to 21)	≥ 10, moderate-to-severe anxiety symptoms [35]	α = 0.84
Posttraumatic Stress Disorder Checklist (PCL) [36]^b^	Posttraumatic Stress Disorder (PTSD)	17	Past month	Likert, 1 (“Not at All”) to 5 (“Extremely”)	Total Score = sum of all 17 items (score range, 0 to 85)	> 50, clinically significant traumatic stress [37]	α = 0.87
**Substance Use**							
Drug Abuse Screening Test-10 (DAST-10) [38]^c^	Drug Use	10	Past 12 months	Dichotomous, 1 (“Yes”) or 0 (“No”)	Total Score = sum of all 10 items (score range, 0 to 10)	≥ 3, risk for drug abuse or dependence [38]	α = 0.58
Alcohol Use Disorders Identification Test-Consumption (AUDIT-C) [39]^d^	Alcohol Consumption	3	None indicated	Likert, 0 (reflects little or no alcohol use) to 4 (reflects high alcohol use)	Total Score = sum of all three items (score range, 1 to 12)	≥ 3 for females and ≥ 4 for males, risk for alcohol abuse or dependence [40]	α = 0.64

^a^In part of the study analyses, scores obtained by summing the first 8 items of the PHQ-9 were used to indicate baseline depression severity (referred to as the PHQ-8) and patients who provided any response other than “Not at all” on item 9, which asks about being bothered by “Thoughts that you would be better off dead, or of hurting yourself in some way,” were considered to have suicidal ideation

^b^Patients who screened positive on the 4-item Primary Care PTSD screening tool (PC-PTSD) [29], indicated by endorsement of two or more symptoms, were determined to be at risk for PTSD and administered the PCL

^c^Patients who screened positive on the 1-item substance use screener [30] were administered the DAST-10

^d^Patients who provided any response other than “Never” on item 1 of the AUDIT-C (“How often do you have a drink containing alcohol?”) were administered the remaining two items

#### Patient characteristics

Self-reported gender, ethnicity, race, and marital status were collected from patients during a clinical visit and recorded in the EHR. Patients had the option to update these characteristics during subsequent visits. Date of birth was also collected and used along with the date of the patient’s baseline visit at a BHA clinic to calculate age at baseline. We followed guidelines established by the United States Census Bureau to categorize race responses [43].

#### Service utilization

Visit information, including visit date and provider type, was recorded in the EHR by BHA staff. Only visits marked as “Completed” were analyzed. Provider type values were re-categorized by mapping Therapist, Social Worker, and Care Coordinator to “Therapist.” Total number of sessions was calculated by counting the number of visits in the first 6 months of treatment.

### Statistical analyses

We first assessed 6-month treatment outcome by identifying whether patients had recorded a PHQ-9 score < 10 (“response”) or a PHQ-9 score < 5 (“remission”) and patients who experienced a ≥ 6 point reduction in PHQ-9 score at least once any time in the first 6 months since their baseline visit. We then used chi-square tests for independence for categorical variables, and two-sample t-tests for continuous variables, to determine any significant differences in demographic and clinical characteristics, and baseline behavioral health conditions, between patients who did and did not demonstrate each treatment outcome. Significant associations were used to identify potential confounding factors controlled for in subsequent analyses.

**Fig. 2:**
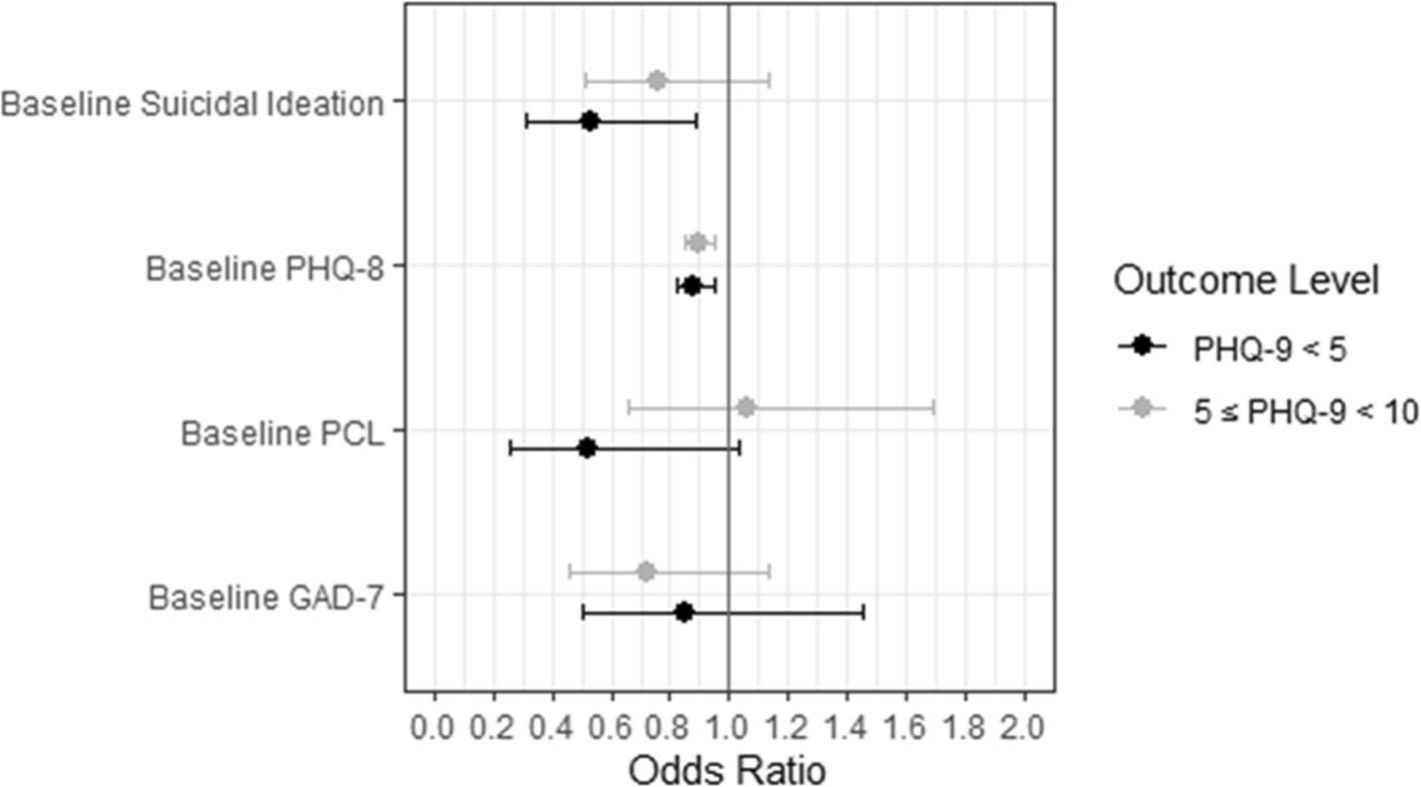
Multinomial logistic regression odds ratios for continued elevated (PHQ-9 ≥ 10), response (5 ≤ PHQ-9 ≤ 9) and remission (PHQ-9 < 5) in the first 6 months, including all baseline behavioral health conditions

To test the hypothesis that the presence of comorbid baseline behavioral health symptoms affects the odds of demonstrating response or remission from depression, we fit multinomial logistic regression models (Fig. 2) with three outcome categories indicating the level of patients’ lowest-recorded PHQ-9 score in the six-months since baseline: a PHQ-9 score < 5 (remission), a PHQ-9 score ≥ 5 and < 10 (response but not remission), or a PHQ-9 score ≥ 10 (maintaining elevated depression symptoms). Creating a 3-level categorical variable and modeling it using a multinomial framework was chosen instead of alternative approaches (for instance, treating the score continuously) because we felt the results from the multinomial models would have greater utility for practitioners. As mentioned previously, BHA clinicians are expected to practice measurement-based-treatment-to-target with targets often specified in terms of response and remission. The multinomial approach also allows for distinct characterizations of factors associated with response and remission rather than constraining these relationships to be ordinal in nature, something that was important to practitioners who posited potentially different factors significantly impacting response and remission.

We fit three separate multinomial models to evaluate the differential impact of baseline suicidal ideation, baseline anxiety, and baseline traumatic stress individually on treatment outcome, while controlling for the severity of baseline depression symptoms. For this and following analyses, we separated item-9 from the PHQ-9 to obtain baseline suicidal ideation and used baseline PHQ-8 scores to measure baseline depression symptoms.

To evaluate the specific impact of each behavioral health symptom on treatment response and remission while controlling for other baseline behavioral health symptoms, we fit a multinomial logistic regression model with the same three outcome categories (remission, response but not remission, and maintaining elevated depression symptoms) with baseline PHQ-8 score, suicidal ideation, baseline anxiety, and baseline traumatic stress as covariates in the same model. The outcome category corresponding to maintaining elevated depression symptoms was used as the reference. We refer to this model as the *co-adjusted* model. Lastly, we fit a multinomial logistic regression model with two additional covariates: an indicator of whether > 50% of a patient’s visits in the first 6 months were to a physician, and the total number of patient visits in the first 6 months. We construct this *fully adjusted* model in an attempt to control for the confounding effects of treatment factors to better understand the prognostic association between baseline behavioral health symptoms and treatment outcomes.

An analogous set of models were constructed to evaluate the impact of specific behavioral health symptoms on demonstration of reliable change. Instead of multinomial logistic regression, we used binary logistic regression to model the probability of experiencing a ≥ 6 point reduction in PHQ-9 score. Analyses were conducted using SAS, Version 9.4 and R, Version 3.6.

## Results

Demographic and behavioral health characteristics for the 615 patients in the analytical sample are displayed in Table 2. Patients were majority female (66%), single (57%), White/Caucasian (58%), and Not Hispanic/Latino (74%). At baseline, behavioral health co-morbidities were substantial with 71% reporting clinical symptoms of anxiety, 40% indicating suicidal ideation, and 28% reporting clinical symptoms of traumatic stress. During the 6 months following each patient’s baseline visit, patients attended a mean of 7.29 (standard deviation = 4.04) treatment sessions. By 6 months, 47% of patients demonstrated response to treatment, 16% demonstrated remission, and 45% experienced a ≥ 6 point reduction in PHQ-9 score.

**Table 2:** Demographics among patients with elevated depression symptoms at baseline (and at least 2 PHQ-9 scores in the first 6 months)

Demographics (***N*** = 615)	N (%)
**Gender**	
Male	211 (34.31)
Female	404 (65.69)
**Ethnicity**	
Hispanic/Latino	99 (16.10)
Not Hispanic/Latino	458 (74.47)
Missing	58 (9.43)
**Race**	
White/Caucasian	354 (57.56)
Asian	54 (8.78)
Black/African American	35 (5.69)
Two or More Races	19 (3.09)
Other/Unknown^a^	153 (24.88)
**Marital Status**	
Married/Domestic Partner	209 (33.98)
Previously Married/Domestic Partner	40 (6.50)
Single	348 (56.59)
Other^b^	18 (2.93)
**Age**	
Age at Baseline, Mean (SD)	40.51 (14.82)
**Baseline Depression Symptoms**	
PHQ-9 Score, Mean (SD)	15.87 (4.56)
**Suicidal Ideation at Baseline**	
Yes^c^	248 (40.33)
**Treatment Response by 6-month Follow-up**	
PHQ-9 Score < 5	100 (16.26)
PHQ-9 Score < 10	289 (46.99)
Reduction in PHQ-9 Score ≥ 6	277 (45.04)
**Other Clinical Symptoms at Baseline**^d,e^	
GAD-7 Score ≥ 10	434 (70.57)
AUDIT-C Score ≥ 3^f^	228 (37.07)
DAST-10 Score ≥ 3^g^	64 (10.41)
PCL Score > 50	174 (28.29)
**Number of Treatment Sessions**	
Total, Mean (SD)^h^	7.29 (4.04)
With a Physician, Mean (SD)	2.81 (2.02)
With a Therapist, Mean (SD)	3.91 (4.33)

^a^ “Other/Unknown” includes: “American Indian or Alaska Native”, “Native Hawaiian or Other Pacific Islander”, “Other”, “Unknown”

^b^ “Other” includes: Life Partner, Significant Other, Unknown, Other, Missing

^c^ Includes any endorsement on PHQ-9 item 9 except “Not at all”

^d^ Percentage is out of the total *N* = 615, though some patients had a missing value on the AUDIT-C, DAST-10, and/or PCL

^e^ For the purposes of the analyses, patients who did not meet screening criteria for a given measure were given a “No” on meeting a clinical threshold (rather than given a missing status)

^f^ Clinical cutoff is ≥3 for females, ≥ 4 for males

^g^ Cutoff ≥3 indicates at least “moderate” symptoms; patients were only administered the DAST-10 if they screened positive on the 1-item screener

^h^ Includes visits that did not indicate a provider type

Using two-group comparisons, we found significant associations between response and remission, and numerous baseline behavioral health symptoms (Table 3). Patients who demonstrated response in the first 6 months had significantly lower depression symptoms at baseline compared to patients who did not demonstrate response (mean diff. = 2.70; *p* < 0.0001). The same was true for remission (mean diff. = 2.31; *p* < 0.0001). Baseline anxiety was also significantly associated with response and remission, with 63% of patients reporting elevated anxiety symptoms at baseline among those who exhibited response, compared to 78% of patients without response (*p* < 0.0001). The prevalence of baseline anxiety was 61% among patients demonstrating remission, compared to 73% among patients who did not demonstrate remission (*p* = 0.0185). Baseline behavioral health symptoms were not associated with experiencing a ≥ 6 point reduction in PHQ-9 score; the exception being baseline depression symptoms. Patients who experienced a ≥ 6 point reduction in PHQ-9 score had significantly higher depression symptoms at baseline compared to patients who did not (mean diff. = 1.35; *p* = 0.0002).

**Table 3:** Demographic and clinical factors for patients by depression symptom outcome at 6-months^a^

	PHQ-9 < 5	PHQ-9 ≥ 5		PHQ-9 < 10	PHQ-9 ≥ 10		Δ PHQ-9 ≥ 6	Δ PHQ-9 < 6	
	N^b^ (%^c^)								
**Baseline PHQ-9**									
Total Score, Mean (SD)	13.94 (3.95)	16.25 (4.58)	**^d^	14.44 (4.08)	17.14 (4.60)	**	16.61 (4.57)	15.26 (4.47)	**
Item 9^e^	25 (25.00)	223 (43.30)	**	91 (31.49)	157 (48.16)	**	114 (41.16)	134 (39.64)	
**Gender**									
Male	32 (32.00)	179 (34.76)		103 (35.64)	108 (33.13)		98 (35.38)	113 (33.43)	
Female	68 (68.00)	336 (65.24)		186 (64.36)	218 (66.87)		179 (64.62)	225 (66.57)	
**Ethnicity**									
Hispanic/Latino	15 (15.00)	84 (16.31)		50 (17.30)	49 (15.03)		47 (16.97)	52 (15.38)	
Not Hispanic/Latino	77 (77.00)	381 (73.98)		215 (74.39)	243 (74.54)		203 (73.29)	255 (75.44)	
Missing	8 (8.00)	50 (9.71)		24 (8.30)	34 (10.43)		27 (9.75)	31 (9.17)	
**Race**									
White/Caucasian	53 (53.00)	301 (58.45)		169 (58.48)	185 (56.75)		163 (58.84)	191 (55.51)	
Asian	9 (9.00)	45 (8.74)		24 (8.30)	30 (9.20)		24 (8.66)	30 (8.88)	
Black/African American	4 (4.00)	31 (6.02)		13 (4.50)	22 (6.75)		11 (3.97)	24 (7.10)	
Two or More Races	5 (5.00)	14 (2.72)		10 (3.46)	9 (2.76)		7 (2.53)	12 (3.55)	
Other/Unknown^f^	29 (29.00)	124 (24.08)		73 (25.26)	80 (24.54)		72 (25.99)	81 (23.96)	
**Marital Status**^g^									
Married/Domestic Partner	41 (41.00)	168 (32.62)		106 (36.68)	103 (31.60)		97 (35.02)	112 (33.14)	
Previously Married/Previous Domestic Partner	6 (6.00)	34 (6.60)		15 (5.19)	25 (7.67)		15 (5.42)	25 (7.40)	
Single	49 (49.00)	299 (58.06)		160 (55.36)	188 (57.67)		154 (55.60)	194 (57.40)	
Other^h^	4 (4.00)	14 (2.72)		8 (2.77)	10 (3.07)		11 (3.97)	7 (2.07)	
**Age**									
Age at Baseline, Mean (SD)	39.64 (14.39)	40.67 (14.91)		40.00 (14.43)	40.95 (15.17)		39.71 (14.27)	41.15 (15.24)	
**Number of Treatment Sessions**^i^									
Total^j^, Mean (SD)	6.96 (4.02)	7.36 (4.05)		7.16 (4.04)	7.41 (4.04)		6.99 (3.94)	7.54 (4.12)	
With a Physician^k^, Mean (SD)	2.48 (2.20)	2.88 (1.98)		2.65 (2.05)	2.95 (2.00)		2.69 (2.08)	2.91 (1.97)	
With a Therapist^l^, Mean (SD)	3.86 (4.38)	3.92 (4.33)		3.96 (4.43)	3.87 (4.26)		3.76 (4.36)	4.03 (4.31)	
**At Risk for Other Behavioral Health Conditions**									
Baseline GAD-7 Score ≥ 10	61 (61.00)	373 (72.71)	*	181 (62.63)	253 (78.09)	**	200 (72.20)	234 (69.64)	
Baseline AUDIT-C Score ≥ 3^m^	31 (31.00)	197 (38.48)		106 (36.81)	122 (37.65)		97 (35.14)	131 (38.99)	
Baseline DAST-10 Score ≥ 3^n^	11 (11.00)	53 (10.29)		25 (8.65)	39 (11.96)		27 (9.75)	37 (10.95)	
Baseline PCL Score > 50^o^	13 (13.00)	161 (31.57)	**	59 (20.56)	115 (35.60)	**	83 (30.07)	91 (27.25)	

^a^ Using 180 days (since baseline BHC) as 6-month indicator

^b^ Sample size including only patients who have at least 2 PHQ-9 scores: *N* = 615; PHQ-9 < 5: *n* = 100; PHQ-9 ≥ 5: *n* = 515; PHQ-9 < 10: *n* = 289; PHQ-9 ≥ 10: *n* = 326; Δ PHQ-9 ≥ 6: *n* = 277; Δ PHQ-9 < 6: *n* = 338

^c^ Percentages displayed represent “column” percentages

^d^ *p-value < 0.05, **p-value < 0.01; for continuous items, *p*-values represent two-sample t-test comparing mean scores between patients who have and have not reached “remission”; for categorical items, *p*-values represent chi-square tests under the null hypothesis of independence between “remission” status and the categorical variable

^e^ Includes any endorsement except “Not at all”

^f^ “Other/Unknown” includes: “American Indian or Alaska Native”, “Native Hawaiian or Other Pacific Islander”, “Other”, “Unknown”

^g^ Significance test

^h^ “Other” includes: Life Partner, Significant Other, Unknown, Other, Missing

^i^ Visits to BHA in the first 6 months

^k^ Includes visits that did not indicate a provider type

^l^ Includes: Physician, Fellow

^m^ Includes: Therapist, Social Worker, Care Coordinator

^n^ Clinical cutoff is ≥3 for females, ≥ 4 for males; patients were only administered the full AUDIT-C if they did not select “never” on the first item of the AUDIT-C

^o^ Cutoff ≥3 indicates at least “moderate” symptoms; patients were only administered the DAST-10 if they screened positive on the 1-item screener

^p^ Patients were only administered the PCL if they did not select “no” for more than one item on the PC-PTSD screen

Baseline suicidal ideation and traumatic stress were significantly lower among patients who demonstrated response and among those demonstrating remission compared to patients who did not demonstrate such improvements. Notably, 25% of patients demonstrating remission reported suicidal ideation at their baseline visit, compared to 43% of patients who did not (*p* = 0.0006), while 31% of patients demonstrating response reported any suicidal ideation at baseline compared to almost one-half (48%) of patients who did not demonstrate response (*p* < 0.0001). Traumatic stress was also less prevalent among patients who reached remission (13%) compared to patients who did not (32%, *p* = 0.0002) and among patients who demonstrated response (21%) compared to patients who maintained elevated depression symptoms (36%, *p* < 0.0001).

Multinomial regression models were estimated for the comorbid behavioral health conditions identified as significant in Table 3. Results indicate significant associations between baseline suicidal ideation and traumatic stress, and remission, even when controlling for baseline depression symptom severity (Table 4). Specifically, the odds of reaching remission were significantly lower among patients with suicidal ideation and patients with elevated traumatic stress. The odds of remission among patients who reported suicidal ideation at baseline was 0.49 times the odds of patients who did not report suicidal ideation at baseline. The odds of remission among patients with a baseline PCL score >  50 was 0.45 times the odds of remission among patients with a baseline PCL score ≤ 50. There is no significant association between suicidal ideation or elevated traumatic stress and the probability of attaining *response but not remission* (PHQ-9 score ≥ 5 and < 10). Baseline depression symptoms are strongly associated with the odds of both *response but not remission* and remission in all models.

**Table 4:** Multinomial logistic regression models for continued elevated (PHQ-9 ≥ 10), response (5 ≤ PHQ-9 ≤ 9) and remission (PHQ-9 < 5) in the first 6 months, controlling for baseline PHQ-8 score

Effect	PHQ-9 Outcome^**a**^	OR	95% CI	***p***-value
Baseline Item 9 (Yes vs. No)	**PHQ-9 < 5**	0.49	(0.29, 0.82)	0.0070
	**5 ≤ PHQ-9 < 10**	0.75	(0.51, 1.10)	0.1406
Baseline PHQ-8 Score	**PHQ-9 < 5**	0.86	(0.80, 0.91)	< 0.0001
	**5 ≤ PHQ-9 < 10**	0.89	(0.84, 0.93)	< 0.0001
Baseline GAD-7 Score ≥ 10	**PHQ-9 < 5**	0.72	(0.43, 1.22)	0.2215
	**5 ≤ PHQ-9 < 10**	0.72	(0.47, 1.10)	0.1300
Baseline PHQ-8 Score	**PHQ-9 < 5**	0.85	(0.80, 0.91)	< 0.0001
	**5 ≤ PHQ-9 < 10**	0.89	(0.85, 0.94)	< 0.0001
Baseline PCL Score > 50	**PHQ-9 < 5**	0.45	(0.23, 0.88)	0.0202
	**5 ≤ PHQ-9 < 10**	0.94	(0.60, 1.47)	0.7738
Baseline PHQ-8 Score	**PHQ-9 < 5**	0.87	(0.81, 0.93)	< 0.0001
	**5 ≤ PHQ-9 < 10**	0.88	(0.84, 0.93)	< 0.0001
*CO-ADJUSTED MODEL*^b^				
Baseline PHQ-8 Score	**PHQ-9 < 5**	0.88	(0.82, 0.95)	0.0004
	**5 ≤ PHQ-9 < 10**	0.90	(0.85, 0.95)	< 0.0001
Baseline Item 9 (Yes vs. No)	**PHQ-9 < 5**	0.53	(0.31, 0.89)	0.0172
	**5 ≤ PHQ-9 < 10**	0.76	(0.51, 1.13)	0.1730
Baseline GAD-7 Score ≥ 10	**PHQ-9 < 5**	0.85	(0.50, 1.45)	0.5561
	**5 ≤ PHQ-9 < 10**	0.72	(0.46, 1.13)	0.1495
Baseline PCL Score > 50	**PHQ-9 < 5**	0.52	(0.26, 1.03)	0.0594
	**5 ≤ PHQ-9 < 10**	1.06	(0.66, 1.69)	0.8178
*FULLY ADJUSTED MODEL*^c^				
Baseline PHQ-8 Score	**PHQ-9 < 5**	0.88	(0.82, 0.94)	0.0003
	**5 ≤ PHQ-9 < 10**	0.89	(0.85, 0.94)	< 0.0001
Baseline Item 9 (Yes vs. No)	**PHQ-9 < 5**	0.53	(0.31, 0.89)	0.0169
	**5 ≤ PHQ-9 < 10**	0.76	(0.51, 1.13)	0.1740
Baseline GAD-7 Score ≥ 10	**PHQ-9 < 5**	0.87	(0.51, 1.48)	0.6038
	**5 ≤ PHQ-9 < 10**	0.72	(0.46, 1.12)	0.1485
Baseline PCL Score > 50	**PHQ-9 < 5**	0.51	(0.26, 1.01)	0.0529
	**5 ≤ PHQ-9 < 10**	1.05	(0.66, 1.67)	0.8482

^a^Relative to PHQ-9 ≥ 10

^b^Adjusted for Baseline PHQ-8 Score, Item 9, GAD-7 Score, and PCL Score

^c^Adjusted for Baseline PHQ-8 Score, Item 9, GAD-7 Score, and PCL Score, Majority Physician Visits, and Total Visit Count

No notable differences were observed between the co-adjusted and fully adjusted multinomial logistic regression models (Table 4). In the fully adjusted model accounting for all comorbid behavioral health conditions and treatment factors, we see the persistence of suicidal ideation as significantly associated with remission. Similar to results from the individual models, there are no significant associations between *response but not remission* and any baseline behavioral health symptoms in the fully adjusted model. The severity of baseline depression symptoms continues to be significantly associated with both response and remission, even after controlling for baseline suicidal ideation, anxiety, and traumatic stress. A one-unit increase in baseline PHQ-8 score is associated with a 11% decrease in the odds of *response but not remission*, and a 12% decrease in the odds of remission in the first 6 months of treatment. The odds of remission among patients with suicidal ideation is 0.53 times the odds among patients without suicidal ideation, when controlling for baseline PHQ-8 score, traumatic stress, and anxiety. The odds of remission among patients with traumatic stress is 0.51 (95% CI = [0.26, 1.01]) times the odds among patients without traumatic stress, when controlling for baseline PHQ-8 score, suicidal ideation, anxiety, majority physician visits and total visit count.

Results from the analogous set of models constructed using the binary outcome corresponding to a ≥ 6 point reduction in PHQ-9 score are displayed in Table 5. Again, there are no notable differences between the co-adjusted and fully adjusted models. Estimated odds ratios are directionally similar to those obtained when examining the response and remission outcomes but confidence intervals are generally wider and none of the comorbid behavioral health effects are statistically significant at the *p* < 0.05 level. Baseline PHQ-8 score is significantly associated with the odds of reliable change across all models. For this outcome, a one-unit increase in baseline PHQ-8 score is associated with a 10% *increase* in the odds of experiencing a ≥ 6 point reduction in PHQ-9 score (fully adjusted model).

**Table 5:** Binary logistic regression models for ≥6 point reduction in PHQ-9 score in the first 6 months, controlling for baseline PHQ-8 score

Effect	PHQ-9 Outcome^**a**^	OR	95% CI	***p***-value
Baseline Item 9 (Yes vs. No)	**Δ PHQ-9 ≥ 6**	0.86	(0.61, 1.22)	0.3964
Baseline PHQ-8 Score	**Δ PHQ-9 ≥ 6**	1.09	(1.05, 1.14)	< .0001
Baseline GAD-7 Score ≥ 10	**Δ PHQ-9 ≥ 6**	0.83	(0.56, 1.22)	0.3313
Baseline PHQ-8 Score	**Δ PHQ-9 ≥ 6**	1.09	(1.05, 1.14)	< .0001
Baseline PCL Score > 50	**Δ PHQ-9 ≥ 6**	0.80	(0.54, 1.20)	0.2800
Baseline PHQ-8 Score	**Δ PHQ-9 ≥ 6**	1.10	(1.05, 1.14)	< .0001
*CO-ADJUSTED MODEL*^b^				
Baseline PHQ-8 Score	**Δ PHQ-9 ≥ 6**	1.10	(1.05, 1.16)	< .0001
Baseline Item 9 (Yes vs. No)	**Δ PHQ-9 ≥ 6**	0.89	(0.63, 1.26)	0.5041
Baseline GAD-7 Score ≥ 10	**Δ PHQ-9 ≥ 6**	0.87	(0.59, 1.30)	0.4940
Baseline PCL Score > 50	**Δ PHQ-9 ≥ 6**	0.85	(0.56, 1.28)	0.4229
*FULLY ADJUSTED MODEL*^c^				
Baseline PHQ-8 Score	**Δ PHQ-9 ≥ 6**	1.10	(1.05, 1.15)	< .0001
Baseline Item 9 (Yes vs. No)	**Δ PHQ-9 ≥ 6**	0.89	(0.62, 1.26)	0.5017
Baseline GAD-7 Score ≥ 10	**Δ PHQ-9 ≥ 6**	0.88	(0.59, 1.31)	0.5382
Baseline PCL Score > 50	**Δ PHQ-9 ≥ 6**	0.83	(0.55, 1.26)	0.3837

^a^ Δ PHQ-9 ≥ 6 indicates a reduction of 6 or greater on the PHQ-9 relative to baseline

^b^Adjusted for Baseline PHQ-8 Score, Item 9, GAD-7 Score, and PCL Score

^c^Adjusted for Baseline PHQ-8 Score, Item 9, GAD-7 Score, and PCL Score, Majority Physician Visits, and Total Visit Count

## Discussion

This study aimed to contribute to knowledge in the field through enhanced understanding of factors that influence depression response and remission, with consideration for design of treatment services to optimize depression outcomes within integrated care programs. Within this study, referred primary care patients endorsed significant symptoms of depression, with a mean PHQ-9 score of 15.87. Strikingly, on intake 40.33% of patients answered affirmatively on PHQ-9 item 9. Anxiety symptoms were highly prevalent (70.57% with GAD-7 Score ≥ 10), 37.07% of patients endorsed at-risk alcohol use (AUDIT-C Score ≥ 3 for females and ≥ 4 for males), 10.41% endorsed at-risk drug use (DAST-10 Score ≥ 3), and 28.29% of patients had a PCL Score >  50, indicating risk for clinically significant trauma symptoms and possible diagnosis of post-traumatic stress disorder. At 6 months, 47 and 16% of patients demonstrated response and remission, respectively. As hypothesized, improved depression treatment outcomes were be associated with fewer baseline behavioral health comorbid symptoms of anxiety and post-traumatic stress. Lack of endorsement of suicidal ideation on intake was associated with improved treatment outcomes. Baseline trauma symptoms and suicidal ideation were significantly associated with decreased odds of achieving remission (Odds Ratio [OR] = 0.45 (0.23, 0.88) and OR = 0.49 (0.29, 0.82), respectively). In fully adjusted models, baseline suicidal ideation remained significant (OR = 0.53 (0.31, 0.89)). Interestingly, substance use was not significantly associated with treatment outcomes.

Depression treatment can be examined in terms of a patient’s achievement of response or remission from symptoms and time to treatment outcome. Given the emphasis of the short-term treatment model of the BHA program and increasing emphasis on national quality outcomes monitoring in depression [13], we focused on treatment outcomes for depression at 6-months of treatment. Similar to previous research, patients who demonstrated response and remission from depression symptoms at 6-months had lower mean depression symptoms on intake. More severe depressive symptoms are associated with poorer functioning and quality of life [44] which are both a sequalae of depression and impact patients’ treatment outcomes [22]. Unsurprisingly, the severity of baseline depression symptoms is significantly associated with both response and remission, even after controlling for baseline suicidal ideation, anxiety, and traumatic stress.

The presence of co-morbid behavioral health symptoms at intake appointment was common among patients referred to the BHA program. The presence of elevated anxiety symptoms on intake was associated with lower likelihood of response or remission from depression within 6-months of treatment. Previous research has reported that anxiety symptoms and anxiety disorders are commonly present within primary care settings [45, 46]. Patients with co-morbid depression and anxiety have a decreased likelihood of remission and increased risk of depression and anxiety severity [47, 48]. Past studies have shown those experiencing co-morbidity also have increased impairment in social and occupational functioning and increased rate of suicide attempts than patients not suffering from comorbidity [47, 49, 50].

The presence of suicidal ideation and trauma symptoms are important to consider in the treatment of depression, as they are common in primary care populations [25, 51–53] and may impact depression treatment outcomes, even after controlling for their associations with baseline depression. Suicidal ideation may occur as a symptom of a depressive episode or it may occur apart from a depressive episode [54]. Suicidal ideation may also be precipitated by trauma [55]. Relatedly, depression itself may be present co-morbid to, or develop as a consequence of, trauma [55].

Importantly, patients endorsing suicidal ideation on intake were less likely to achieve depression response or remission. Results revealed the odds of remission among patients with suicidal ideation is 0.53 times the odds among patients without suicidal ideation, when controlling for baseline PHQ-8 score, traumatic stress, and anxiety. This is a particularly interesting finding, given presence of suicidal ideation suggests more severe depressive symptoms (higher initial PHQ-9 scores). According to Pompili [54], there is evidence that suicidality itself may impact treatment response to antidepressant medications, independent of overall depression severity. Further, it is noted that “such evidence seems to suggest that depressed, suicidal individual represent a peculiar subgroup of patients that request in-depth clinical observation” [54]. These results suggest clinicians may find utility in examining suicidality as a separate predictive factor in depression treatment. Patients with suicidality may require more aggressive medication management and therapy. Patients with challenges in emotional regulation and those exhibiting self-harm behaviors may benefit from dialectical behavioral therapy [56, 57]. In this study the rate of endorsement on PHQ-9 item 9 among those referred to the program may partly be attributable to a selection bias, as patients who appeared more depressed to their primary care providers may have been more likely to be referred. However, while taking potential selection bias into consideration, this high rate also underscores the importance of systematic screening for suicidal ideation and safety planning in the population.

On intake, 28.29% of patients had a PCL Score > 50, indicating risk for clinically significant trauma symptoms and possible diagnosis of post-traumatic stress disorder. It has been reported that 2–39% of primary care patients may have a diagnosis of post-traumatic stress disorder (PTSD) [51, 53, 58], with a recent United Kingdom study providing a prevalence estimate of 15.5% [59]. The rate of trauma symptoms reported in this referred population supports these estimates. The presence of trauma symptoms on intake was associated with lower likelihood of remission from depression within 6-months of treatment after controlling for depression symptom severity on intake. Specifically, in terms of impact on depression outcomes, the odds of remission among patients with a baseline PCL score > 50 was 0.45 times the odds of remission among patients with a baseline PCL score ≤ 50. Results were similar in the fully adjusted model (odds ratio = 0.51), although not statistically significant. The lower estimated likelihood of remission from depression in these patients indicates that patients with depression should be screened for trauma symptoms. Psychiatric evaluation of this symptom domain will further inform depression treatments and these results suggest the need for potentially greater service intensity (number of treatment sessions, frequency of sessions) and/or more targeted therapies and medication management to address co-morbid trauma symptoms and possible PTSD diagnosis. Treatment programs may explore the implementation of trauma-focused treatments such as cognitive processing therapy, prolonged exposure, or eye movement desensitization and reprocessing therapies [60, 61]. It is important for providers and health systems to understand patient-related factors associated with depression remission and response rates in clinical treatment settings. These factors may be employed to increase specificity of treatment services to help patients achieve remission from depression symptoms.

Highlighting difficulties inherent in using EHR data, we relied on system labels to define baseline and follow-up visits. This includes identifying baseline visits as an appointment status labeled as *New*, with subsequent visits assumed to be corresponding follow-ups. A patient’s true first visit to BHA could have been earlier than defined. We acknowledge the challenges with generalizability of our results due to the racial/ethnic and economic (e.g. insurance status) composition of our patient population. Although we adjusted for baseline depression symptom severity, the potential impact of regression to the mean is a necessary consideration when interpreting these and other study findings based on the modeling of behavioral health symptoms over time. Selection of potential confounders to adjust for was not undertaken in a manner recommended for causal inference. Results are intended to provide insight into the probability of attaining certain treatment outcomes at 6-months regardless of the dose, duration, or type of treatment a patient engages in. While this has clinical utility, it is important to distinguish these findings from those you might publish were intermediary variables related to dose-response strategically considered for inclusion following causal analysis guidelines. Nevertheless, there are many important factors such as symptom chronicity, history of antidepressant treatment, and physical health comorbidities that were unavailable for this study and thus could not be considered for inclusion in the regression models. The potential impact of excluding these factors is unknown but should not be disregarded. While our methodological approach satisfies an evaluation of short-term depression outcomes based on often-used clinical cutoffs, we know that many patients continue in BHA after the six-month mark and that evaluation of their longer-term symptom trajectories could offer valuable insight to patterns of symptoms over time. Future work will explore this topic.

## Conclusion

Increased understanding of factors associated with depression treatment outcomes may be employed to help guide the delivery and design of clinical services. Alongside routine screening for co-morbid anxiety, suicidal ideation and traumatic stress should be assessed and considered when designing depression treatment services.

## Data Availability

The datasets generated and/or analyzed during the current study are not publicly available due to privacy restrictions but are available from the corresponding author on reasonable request.
